# Bone Morphogenetic Protein (BMP)-3b Gene Depletion Causes High Mortality in a Mouse Model of Neonatal Hypoxic-Ischemic Encephalopathy

**DOI:** 10.3389/fneur.2018.00397

**Published:** 2018-06-05

**Authors:** Yuko Ogawa, Masahiro Tsuji, Emi Tanaka, Mikiya Miyazato, Jun Hino

**Affiliations:** ^1^Department of Regenerative Medicine and Tissue Engineering, National Cerebral and Cardiovascular Center, Suita, Japan; ^2^Department of Biochemistry, National Cerebral and Cardiovascular Center, Suita, Japan

**Keywords:** bone morphogenetic protein-3b, neonatal brain injury, hypoxia-ischemia, hypoxic-ischemic encephalopathy, mice, growth and differentiation factor 10

## Abstract

Bone morphogenetic proteins (BMPs) are a group of proteins that induce the formation of bone and the development of the nervous system. BMP-3b, also known as growth and differentiation factor 10, is a member of the BMPs that is highly expressed in the developing and adult brain. BMP-3b is therefore thought to play an important role in the brain even after physiological neurogenesis has completed. BMP-3b is induced in peri-infarct neurons in aged brains and is one of the most highly upregulated genes during the initiation of axonal sprouting. However, little is known about the role of BMP-3b in neonatal brain injury. In the present study, we aimed to describe the effects of BMP-3b gene depletion on neonatal hypoxic-ischemic encephalopathy, which frequently results in death or lifelong neurological disabilities, such as cerebral palsy and mental retardation. BMP-3b knockout and wild type mice were prepared at postnatal day 12. Mice of each genotype were divided into sham-surgery, mild hypoxia-ischemia (HI), and severe HI groups (*n* = 12–45). Mice in the HI groups were subjected to left common carotid artery ligation followed by 30 min (mild HI) or 50 min (severe HI) of systemic hypoxic insult. A battery of tests, including behavioral tests, was performed, and the brain was then removed and evaluated at 14 days after insult. Compared with wild type pups, BMP-3b knockout pups demonstrated the following characteristics. (1) The males exposed to severe HI had a strikingly higher mortality rate, and as many as 70% of the knockout pups but none of the wild type pups died; (2) significantly more hyperactive locomotion was observed in males exposed to severe HI; and (3) significantly more hyperactive rearing was observed in both males and females exposed to mild HI. However, BMP-3b gene depletion did not affect other parameters, such as cerebral blood flow, cylinder test and rotarod test performance, body weight gain, brain weight, spleen weight, and neuroanatomical injury. The results of this study suggest that BMP-3b may play a crucial role to survive in severe neonatal hypoxic-ischemic insult.

## Introduction

Bone morphogenetic proteins (BMPs) form a subgroup in the transforming growth factor-β (TGF-β) superfamily and act as powerful morphogens that perform crucial roles during embryonic development (1, 2). Although they were first discovered as osteoinductive proteins, they were later recognized as critical regulators of nervous system development (2). BMP signaling plays dynamic roles in the development of the brain in the early stages of life, during which they sequentially induce neurogenesis and then astrogliogenesis (3). BMPs also regulate neurite outgrowth from immature neurons during forebrain development and are crucial for maintaining adult neural stem cell niches in the subventricular and subgranular zones (2). Previous studies have suggested that while certain BMPs are beneficial (4–6), other BMPs are detrimental after ischemic brain injury (7, 8).

In 1996, we discovered a protein that was structurally similar to BMPs, especially BMP-3. We therefore named it BMP-3b (9, 10), and it is currently also known as growth and differentiation factor 10 (GDF10) (11). Unlike other BMPs, BMP-3b/GDF10 suppresses osteoblast differentiation by activating Smad2/3 signaling via the ALK4/ActRIIA receptors (12). We previously reported that BMP-3b is essential for head formation in *Xenopus* embryos and acts as a neural inducing factor (13). BMP-3b is strongly expressed in developing skeletal structures in embryos and in bones, the brain (especially the cerebellum), the aorta, and adipose tissues in adult rodents (14, 15). Despite the high level of BMP-3b expression observed in these organs, no obvious abnormality was noted in the development of these tissues and organs in BMP-3b knockout (KO) mice (14). It is conceivable that BMP-3b plays a role in brain injury events because it is highly expressed in the brain throughout life. Recently, Li and colleagues reported that BMP-3b is induced in peri-infarct neurons and that it enhanced axonal sprouting and functional recovery in a mouse model of stroke (16). However, the role of BMP-3b in injury to the developing brain has not yet been explored.

Perinatal/neonatal hypoxic-ischemic encephalopathy (HIE) is the most serious problem encountered in neonatal neurology. HIE occurs in 1–2/1000 births and can be caused by a variety of events, such as abruptio placentae and umbilical cord prolapse. Although the development of therapeutic hypothermia to treat neonatal HIE was a landmark achievement in neonatal care, the mortality rate in HIE patients treated for hypothermia in neonatal intensive care units remains approximately 10% (17). Additionally, many of the infants who survive suffer lifelong neurological sequelae, such as cerebral palsy and mental retardation. Therefore, novel therapies need to be developed.

The aim of the present study was to examine the physiological roles of endogenous BMP-3b in neonatal HIE using mouse models in which BMP-3b has or has not been depleted. Specifically, we focused on the effects of BMP-3b gene depletion on outcomes related to neurological damage.

## Materials and methods

### Animal model of hypoxic-ischemic encephalopathy

All procedures were performed according to protocols approved by the Animal Care and Use Committee of the National Cerebral and Cardiovascular Center. The BMP-3b/GDF10 KO mice were kindly provided by Se-Jin Lee. BMP-3b KO mice were backcrossed with C57BL/6 mice more than 10 times. In all, 41 BMP-3b KO males, 46 BMP-3b KO females, 22 wild type males, and 27 wild type females were prepared. Mouse pups were obtained at postnatal day 12 (P12), which is considered the equivalent of human term newborns at P0. Mice of each genotype were divided into the following four groups: no-surgery control, sham-surgery control, mild hypoxia-ischemia (HI), or severe HI (*n* = 12–45) (Table 1). Littermates were randomly assigned to one of the four groups.

**Table 1:** Numbers of animals prepared and mortality rates in each experimental group.

		**Wild**			**BMP-3b KO**		
		**Prepared**	**Dead**	**Mortality rate**	**Prepared**	**Dead**	**Mortality rate**
		***(n)***	***(n)***	**(%)**	***(n)***	***(n)***	**(%)**
No-surgery	Male				5	0	0
	Female				9	0	0
Sham	Male	6	0	0	7	0	0
	Female	7	0	0	5	0	0
HI-30min	Male	9	0	0	6	0	0
	Female	11	0	0	10	0	0
HI-50min	Male	7	0	0	23	16^a^^,^^b^	70
	Female	9	2	22	22	7	32

BMP-3b KO, bone morphogenetic protein-3b knockout; HI, hypoxia-ischemia.

a After HI-50 min insult, the mortality rate in BMP-3b KO males was significantly higher than the rate in wild type males. p < 0.01.

b *After HI-50 min insult, the mortality rate in BMP-3b KO males was significantly higher than the rate in BMP-3b females. p < 0.05*.

We used the Rice-Vannucci model (18), which is the most widely used animal model of HIE. Under isoflurane anesthesia (4.0% for induction and 1.5 to 2.0% for maintenance), the left carotid artery was separated from the jugular vein and connective tissue, doubly ligated, and then severed between the two ligatures. All the surgeries were performed by a single experimenter to reduce variability between experimenters. After surgery, the mouse pups were allowed to recover for 1–2 h while separated from their dam. In the mice exposed to hypoxia, littermates were placed in an enclosed vented chamber that was flushed with a humidified mixture of 8% oxygen (balance nitrogen) for either 30 min (mild HI) or 50 min (severe HI). Mice in the sham-surgery control group underwent the same procedure except for artery ligation and exposure to hypoxia. The ambient temperature inside the chamber was kept at 33.0°C, which is the temperature the pups are normally exposed to while huddling with their dam. The mice were then allowed to recover for 1 h in an incubator set to an ambient temperature of 33.0°C, and they were then returned to their dams. All analyses were performed by investigators who were blinded to the experimental group.

### Cerebral blood flow measurements

**Figure 1:**
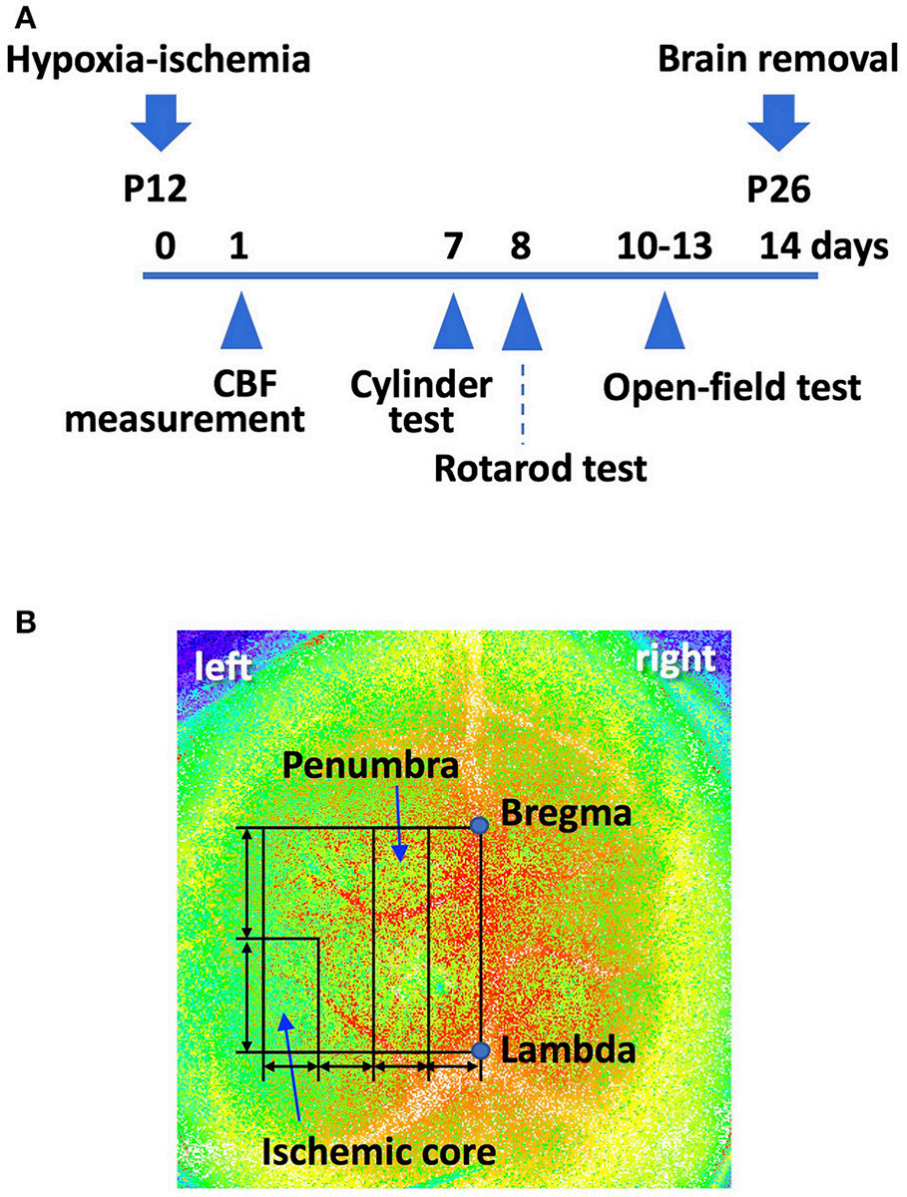
**(A)** A scheme of the experiment schedule. P; postnatal day. **(B)** Using laser speckle flowmetry, we measured cerebral blood flow (CBF) in the “penumbra” and “ischemic core” regions, both of which were set as shown, at 24 h after the surgery. A line was drawn from bregma to lambda. This line was used to draw a square. The line perpendicular to the bregma-lambda line was divided into four equal segments. The second quarter-rectangle from the center was defined as the “penumbra” region. The posterior half of the most lateral segment was defined as the “ischemic core” region. We also measured CBF in the corresponding region of the contralateral hemisphere.

We measured cortical surface cerebral blood flow (CBF) with a laser speckle flowmetry imaging system (Omegazone, Omegawave Inc., Tokyo, Japan). Measurements were obtained through the intact skull and an open scalp at 24 h after the surgery, as previously described (19). We measured CBF in both the ischemic core region and the penumbra region (the broader region between the ischemic and intact regions) (Figures 1A,B). We also measured CBF in the corresponding region of the contralateral hemisphere.

### Behavioral tests

The no-surgery controls were tested only in cylinder and rotarod tests. The data obtained in both the no-surgery and sham-surgery controls were combined because there was no difference in performance between the groups.

#### Cylinder test

Forelimb functional impairment was evaluated in cylinder tests 7 days after the surgery (P19), as previously described (20). We placed each mouse in a transparent cylinder (14 cm inner diameter and 30 cm high) and video recorded its behavior until it reared and touched the cylinder wall with its forepaw(s) a minimum of 20 times. In most mice, this took 3–5 min. We counted the number of contacts with the wall by the left or right forepaw during a rear separately. Paw preference in wall touch (i.e., asymmetry of forelimb use) was calculated using the following formula: (left [non-impaired side]—right [impaired side])/(left + right) × 100%.

#### Rotarod test

Sensorimotor skills were evaluated in rotarod tests 8 days after the surgery (P20). The rotarod was accelerated from 4 to 40 rpm over 5 min (Ugo Basile, Co., Ltd., Gemonio, Italy). We recorded the time until the pup fell off the rotating drum in five consecutive sessions. The average time spent on the drum was used for the analysis.

#### Open-field test

Spontaneous activities and exploratory behaviors were evaluated in open-field tests 10–13 days after the surgery (P22–25), as previously described (21). Each animal was allowed to search freely in a box (30 × 30 cm) for 30 min in a light environment and then for 30 min in a dark environment. Infrared beams were mounted at specific intervals on the X-, Y-, and Z-banks of the open-field box (Taiyo Electric Co., Ltd, Osaka, Japan). The total number of beam crossings made by the mouse was counted and scored as “locomotion” for horizontal movements and “rearing” for vertical movements.

### Neuroanatomical analysis

Fourteen days after surgery, the mice were anesthetized with an overdose of pentobarbital and then perfused via the left ventricle with phosphate-buffered saline followed by 4% paraformaldehyde. After perfusion, the brains of the mice were removed and weighed. After they were placed in the same fixative for 2–3 days, the brains were coronally sectioned into 1 mm-thick slices using a mouse brain slicer (Neuroscience Idea Co., Ltd., Osaka, Japan). Areas in the ipsilateral and contralateral hemispheres of each brain section were measured using the ImageJ program (NIH, Bethesda, MD, USA). Hemispheric volume was estimated by integrating the hemispheric areas. The brains were further thin-sectioned, 6 μm-thick, and stained with hematoxylin-eosin. We evaluated neuropathological injury semi-quantitatively in four brain regions (the cerebral cortex, hippocampus, striatum, and thalamus), as previously described (22). The total score (0–22) was obtained by calculating the sum of the ratings in the four brain regions. We also evaluated white matter injury semi-quantitatively in corpus callosum in 6 μm-thick sections with Klüver-Barrera staining. The severity of white matter injury was scored (0–3) as previously described (23).

### Statistical analysis

The mortality rate of the animals was analyzed with the Fisher's exact test with a Bonferroni correction for multiple comparisons. Data on body, brain, and spleen weights; CBF; cylinder and rotarod tests; and hemispheric volumes were assessed using two-way analysis of variance (ANOVA) followed by a Bonferroni test to analyze the effects of HI insult and genotype. Because the injury scores were not distributed normally, they were assessed with the Kruskal-Wallis test followed by Dunn's multiple comparisons test. Data from the open-field tests were assessed using two-way repeated-measures ANOVA followed by a Bonferroni test. Although data of sham, HI-30 min, and HI-50 min groups were analyzed all together, data of HI-30 min and those of HI-50 min in the open-field test are presented separately with the same data of sham group in order to avoid busy figures. Data of males and females were analyzed and are presented separately. Some of the data, however, are presented males and females combined as there were no significant difference between males and females. Differences were considered significant at *p* < 0.05. Significant differences between groups with the same genotype or that received the same magnitude of insult are presented in this manuscript and figures. Other comparisons, such as comparisons between BMP-3b KO mice treated with sham-surgery and wild type mice treated with severe hypoxia (HI-50 min) insult, are not presented. The results are presented as the mean ± standard deviation (SD) except for data from the open-field tests, which are presented as the mean ± standard error of the mean (SEM).

## Results

### Body weights and mortality rates

**Figure 2:**
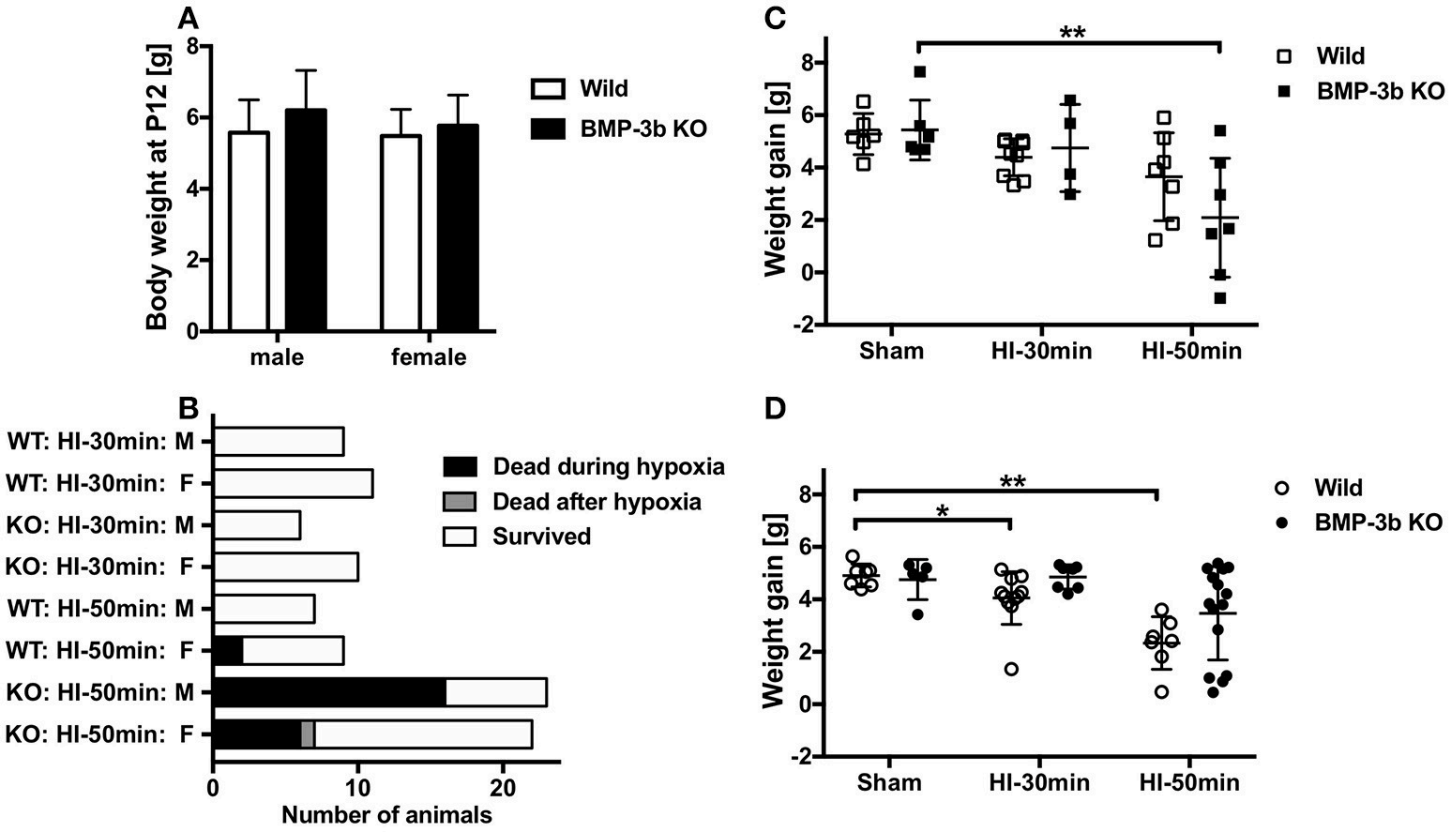
**(A)** Body weights of mouse pups at the time of surgery (postnatal day 12, P12). Bone morphogenetic protein (BMP)-3b knockout (KO) males, *n* = 41; BMP-3b KO females, *n* = 46; wild type males, *n* = 22; and wild type females, *n* = 27. Mean ± standard deviation (SD). **(B)** The numbers of animals dead and survived in each group after the hypoxic-ischemic (HI) insult. One female animal died 6 days after the 50 min HI insult. **(C)** Body weight gain in male pups during the observation period from P12 to P26. **(D)** Body weight gain in female pups during the observation period. Sham, sham-surgery controls; HI-30 min, hypoxia-ischemia for 30 min; HI-50 min, hypoxia-ischemia for 50 min. ^*^p < 0.05, ^**^p < 0.01.

The mean body weight at the time of surgery (P12) was 6.20 ± 1.10 g in BMP-3b KO males, 5.77 ± 0.85 g in BMP-3b KO females, 5.58 ± 0.89 g in wild type males, and 5.48 ± 0.73 g in wild type females (Figure 2A). Body weights did not differ among these groups.

None of the animals subjected to a sham-surgery or the HI-30 min insult died during the 14-day observation period (until P26) regardless of the genotype. In animals subjected to the HI-50 min insult, the mortality rate was 70% in male BMP-3b KO pups, and this was significantly higher than the rate observed in male wild type pups (0%). Almost all deaths occurred during the last 15 min of hypoxic exposure or immediately after it ended. The mortality rate was 32% in female BMP-3b KO pups, and this was not significantly different from the rate observed in female wild type pups (22%) (Table 1,Figure 2B).

In the animals treated with a sham-surgery, body weight gains during the 14-day observation period were similar between wild type pups and BMP-3b KO pups. However, weight gain was lower in the HI-50 min insult groups than in the sham-surgery groups. The magnitude of this reduction did not differ according to genotype. When stratified by sex, BMP-3b KO males but not wild type males had significantly lower body weight gains. In contrast, wild type females but not BMP-3b KO females had significantly lower body weight gains (Figures 2C,D).

### Cerebral blood flow

**Figure 3:**
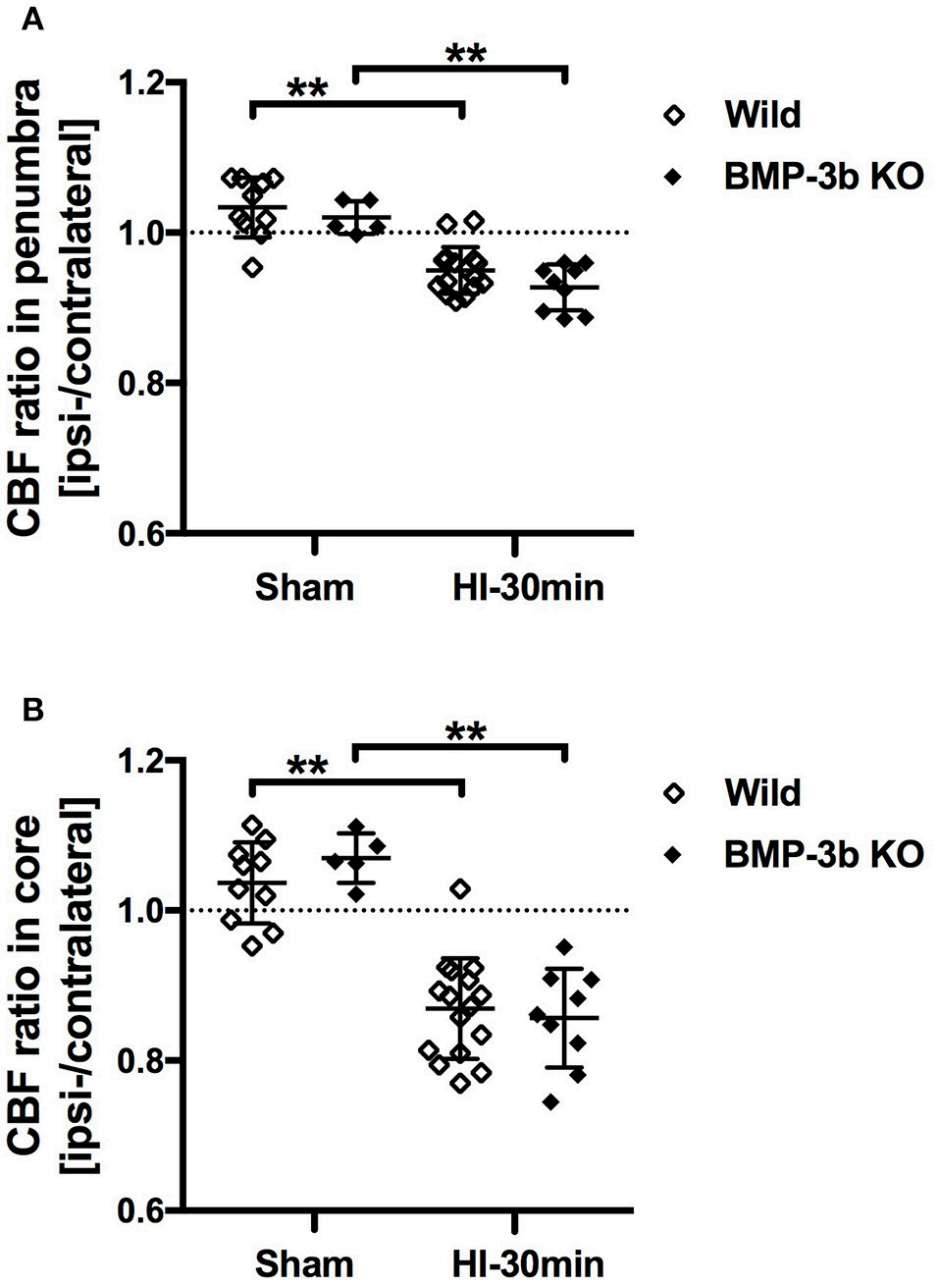
**(A)** The ratios of CBF in the penumbra region of the ipsilateral (left) hemisphere to the corresponding region of the contralateral (right) hemisphere are shown. **(B)** The same CBF ratios in the ischemic core regions. Sham, sham-surgery controls; HI-30 min, hypoxia-ischemia for 30 min. ^**^p < 0.01.

Surface CBF in the right hemisphere (i.e., the contralateral side) was not different between wild type and BMP-3b KO pups at 24 h after sham-surgery [40.0 ± 7.3 and 38.0 ± 6.0 (arbitrary units), respectively]. There was also no significant different in CBF in the contralateral hemisphere between wild type and BMP-3b KO pups at 24 h after HI-30 min insult [43.0 ± 3.6 and 38.1 ± 7.6 (arbitrary units), respectively]. In the ipsilateral hemisphere, CBF was lower after HI insult (Figure 1B). The ratio of CBF in the ischemic core and penumbral regions of the ipsilateral hemisphere to the corresponding regions in the contralateral hemisphere was not different between wild type and BMP-3b KO pups (Figures 3A,B). There was no difference according to gender within each group.

### Cylinder test

**Figure 4:**
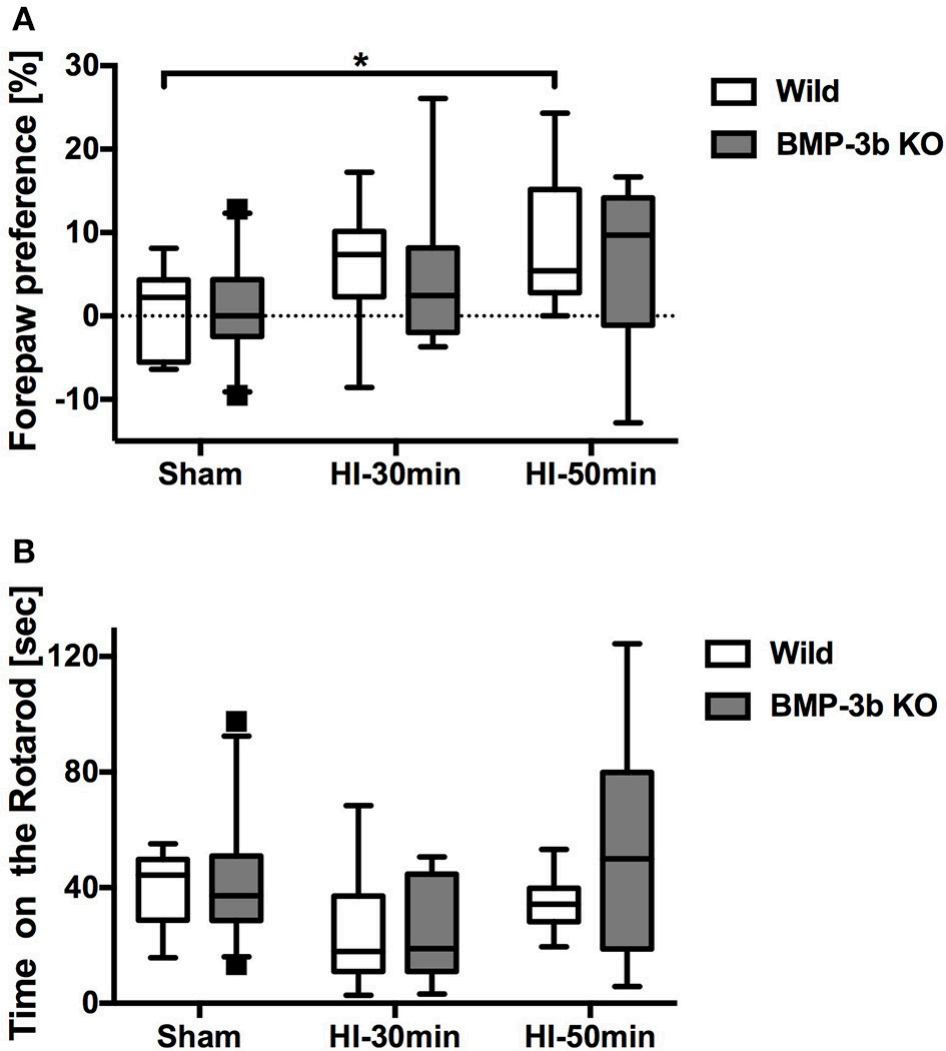
**(A)** Hemiplegia, i.e., disuse of the affected right forepaw, was evaluated in cylinder tests 7 days after surgery (i.e., postnatal day 19, P19). The number of forepaw contacts made with the wall during rearing were counted. Paw preference was calculated as follows: (left [non-impaired side]—right [impaired side])/(left + right) × 100%. **(B)** Sensorimotor skills were evaluated in rotarod tests 8 days after surgery (P20). The average time until the animal fell off the rotating drum in 5 consecutive sessions was analyzed. The box shown extends from the 25 to 75th percentiles. The whiskers shown are drawn down to the 5th and up to the 95th percentile. Points below and above the whiskers are drawn as individual dots. The no-surgery controls were combined with the sham group because there was no difference in performance between these groups. In the cylinder test, Wild Sham, *n* = 12; Wild HI-30 min, *n* = 16; Wild HI-50 min, *n* = 8; BMP-3b KO Sham, *n* = 22; BMP-3b KO HI-30 min, *n* = 12; and BMP-3b KO HI-50 min, *n* = 17. In the rotarod test, Wild Sham, *n* = 7; Wild HI-30 min, *n* = 10; Wild HI-50 min, *n* = 10; BMP-3b KO Sham, *n* = 25; BMP-3b KO HI-30 min, *n* = 12; and BMP-3b KO HI-50 min, *n* = 9. ^*^p < 0.05.

The cylinder test was performed at 7 days after the surgery (P19), and the results showed that motor impairment of the affected forelimb (i.e., the right side) had occurred in pups subjected to HI insult (Figure 4A). While forepaw preferences were significantly different between types of insult, they did not differ by genotype (two-way ANOVA). *Post*-*hoc* tests showed that significant group differences were observed only between the sham and HI-50 min groups among the wild type mice. There was no difference according to sex within any group.

### Rotarod test

Rotarod performance was measured at 8 days after sham-surgery (P20), and no difference was detected between the wild type and BMP-3b KO pups (Figure 4B). However, the Rotarod test results demonstrated that sensorimotor impairment occurred in pups subjected to HI insult. Similar to the results of cylinder tests, there was no difference in time spent on the rotarod according to genotype, whereas they were significantly different according to the type of insult (two-way ANOVA). *Post*-*hoc* tests, however, did not reveal any significant group differences, and there was no difference within each group according to sex.

### Open-field test

**Figure 5:**
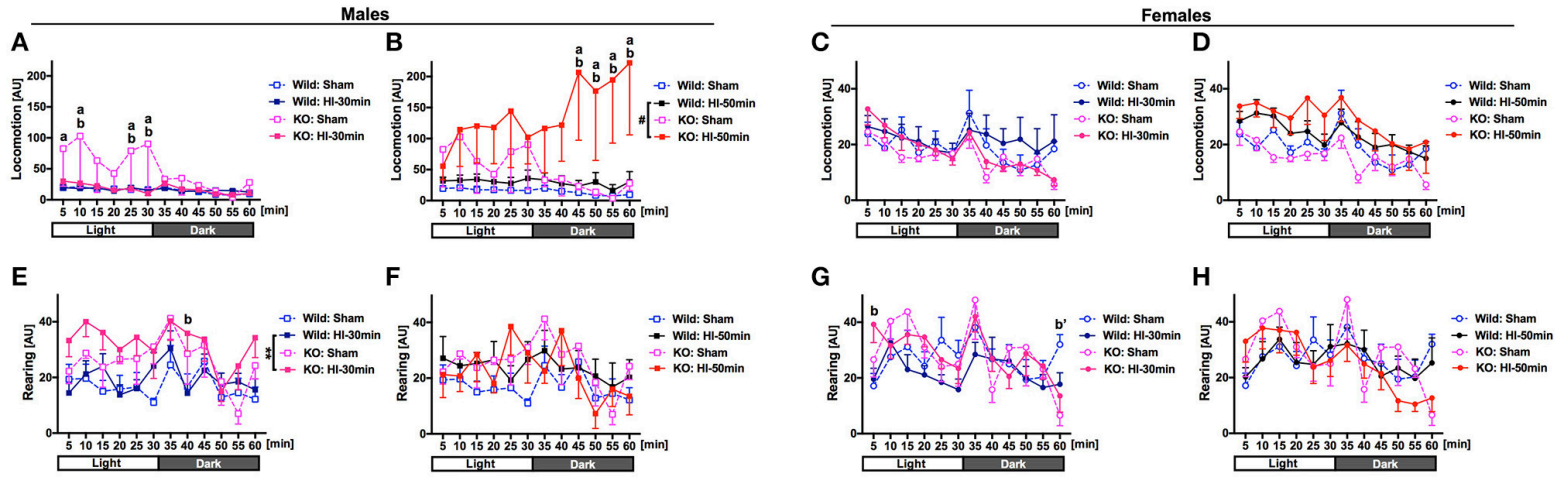
Spontaneous activities were evaluated in open-field tests 10–13 days after surgery (P22-25). Mice were allowed to search freely in a box for 30 min while in a light environment and for the next 30 min while in a dark environment. The total number of times that the infrared beams set at specific intervals along the X-, Y-, and Z-banks of the box were crossed by each animal was counted and scored as “locomotion” for horizontal movement (males: **A,B**, females: **C,D**) and as “rearing” for vertical movement (males: **E,F**, females: **G,H**). The experiments were designed and performed in six groups, i.e., sham, HI-30 min, and HI-50 min groups, each containing both wild type and BMP-3b KO subgroups. To avoid the use of busy graphs with six lines, the HI-30 min and HI-50 min groups are presented separately, each with the same sham group. Mean ± standard error of the mean (SEM). AU, arbitrary unit. Males: Wild Sham, *n* = 6; Wild HI-30 min, *n* = 9; Wild HI-50 min, *n* = 7; BMP-3b KO Sham, *n* = 4; BMP-3b KO HI-30 min, *n* = 7; BMP-3b KO HI-50 min, *n* = 4. Females: Wild Sham, *n* = 7; Wild HI-30 min, *n* = 11; Wild HI-50 min, *n* = 7; BMP-3b KO Sham, *n* = 5; BMP-3b KO HI-30 min, *n* = 9; and BMP-3b KO HI-50 min, *n* = 13. #*p* = 0.08, ^**^p < 0.01. a: *p* < 0.05, KO HI vs. KO Sham. b: *p* < 0.05, KO HI vs. Wild HI. b′: *p* < 0.05, KO Sham vs. Wild Sham.

Spontaneous activities were evaluated in open-field tests performed at 10–13 days after the surgery (P22–25). With regard for locomotion (i.e., horizontal movement), in the animals submitted to sham-surgery, in a light environment, BMP-3b KO males were significantly more hyperactive than the wild type males were (Figure 5A). After HI-50 min insult but not after HI-30 min insult, in a dark environment, BMP-3b KO males became significantly more hyperactive than did the wild type males (Figure 5B). There was no difference between the BMP-3b KO females and wild type females after sham-surgery, HI-30 min insult, or HI-50 min insult (Figures 5C,D).

With regard for rearing (i.e., vertical movement), after the HI-30 min insult, throughout the 60 min-session, including the light and dark environment periods, BMP-3b KO males were significantly more hyperactive than wild type males were (Figure 5E). However, after the HI-50 min insult, BMP-3b KO males were not more hyperactive than wild type males were (Figure 5F). After sham-surgery, BMP-3b KO females were significantly more hypoactive than wild type females during the last 5 min of the 60 min-session (in a dark environment) (Figure 5G). In contrast, after the HI-30 min insult, BMP-3b KO females were significantly more hyperactive than wild type females during the first 5 min of 60 min-session (in a light environment) (Figure 5G). After the HI-50 min insult, BMP-3b KO females did not exhibit significantly different spontaneous activities compared with the wild type females (Figure 5H).

### Brain weight

**Figure 6:**
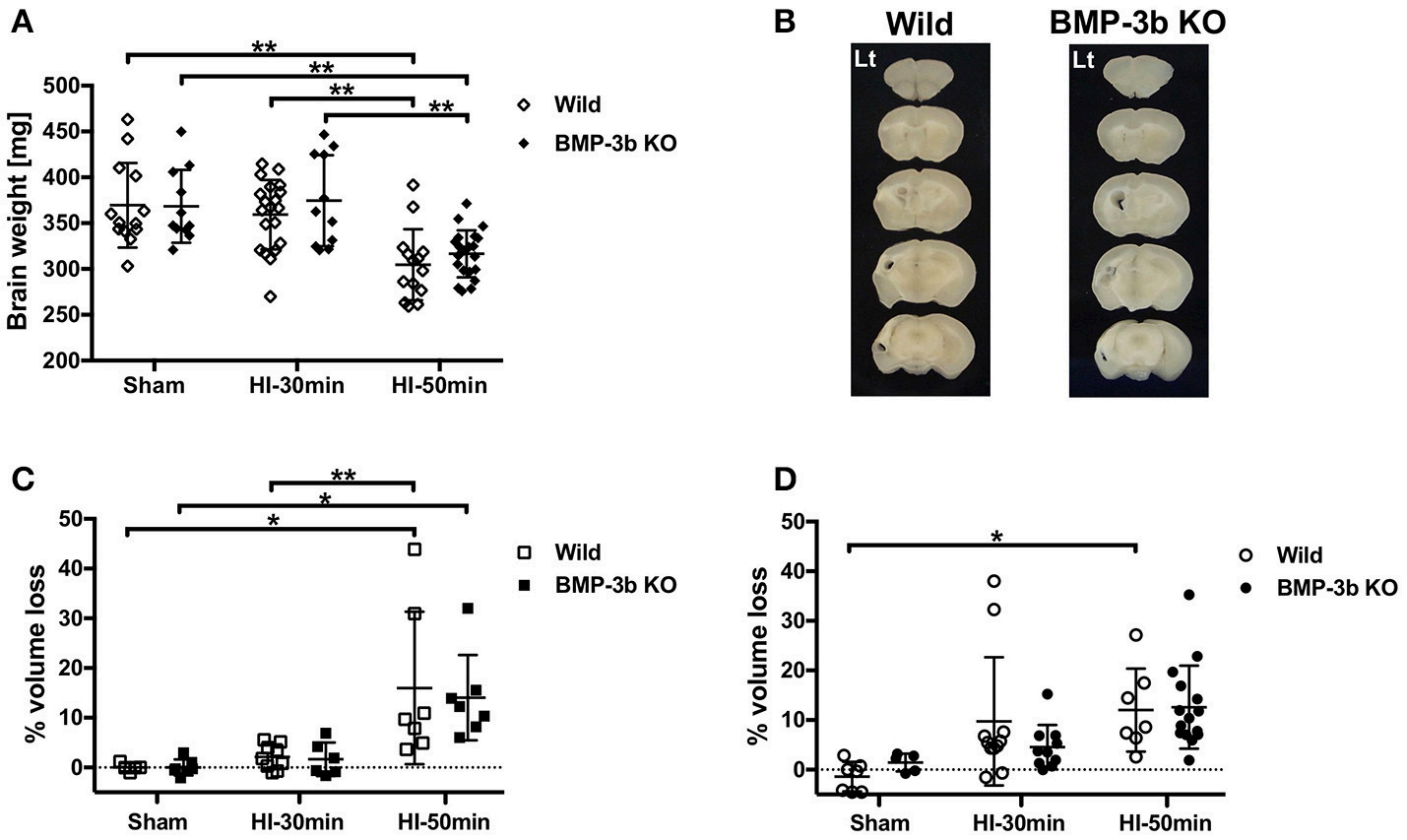
**(A)** Brain weights at 14 days after surgery. **(B)** Representative images of 1-mm slices of brains after HI-50 min insult at 14 days after the surgery. **(C,D)** Percent cerebral hemispheric volume loss: 100 × (contralateral hemispheric volume—ipsilateral hemispheric volume)/contralateral hemispheric volume. Males: **(C)**, females: **(D)**. ^*^p < 0.05, ^**^p < 0.01.

Brain weights were similar between wild type pups and BMP-3b KO pups at 14 days after sham-surgery (P26). The HI-50 min insult but not the HI-30 min insult resulted in lower brain weight at P26 (Figure 6A). There were no genotype-dependent differences between animals treated with the same degree of insult. There were no sex-dependent differences in any genotype or insult.

### Hemispheric volume loss

In animals examined at 14 days after insult, the HI-30 min insult caused a mild loss in hemispheric volume, but the volumes in these animals were not significantly different from those observed in the sham-surgery-treated animals in either males (Figure 6C) or females (Figure 6D). The HI-50 min insult caused a significant loss in hemispheric volume in wild type and BMP-3b KO males and wild type females (Figures 6B–D). There were no significant genotype- or sex-dependent differences.

### Neuropathological injury

**Figure 7:**
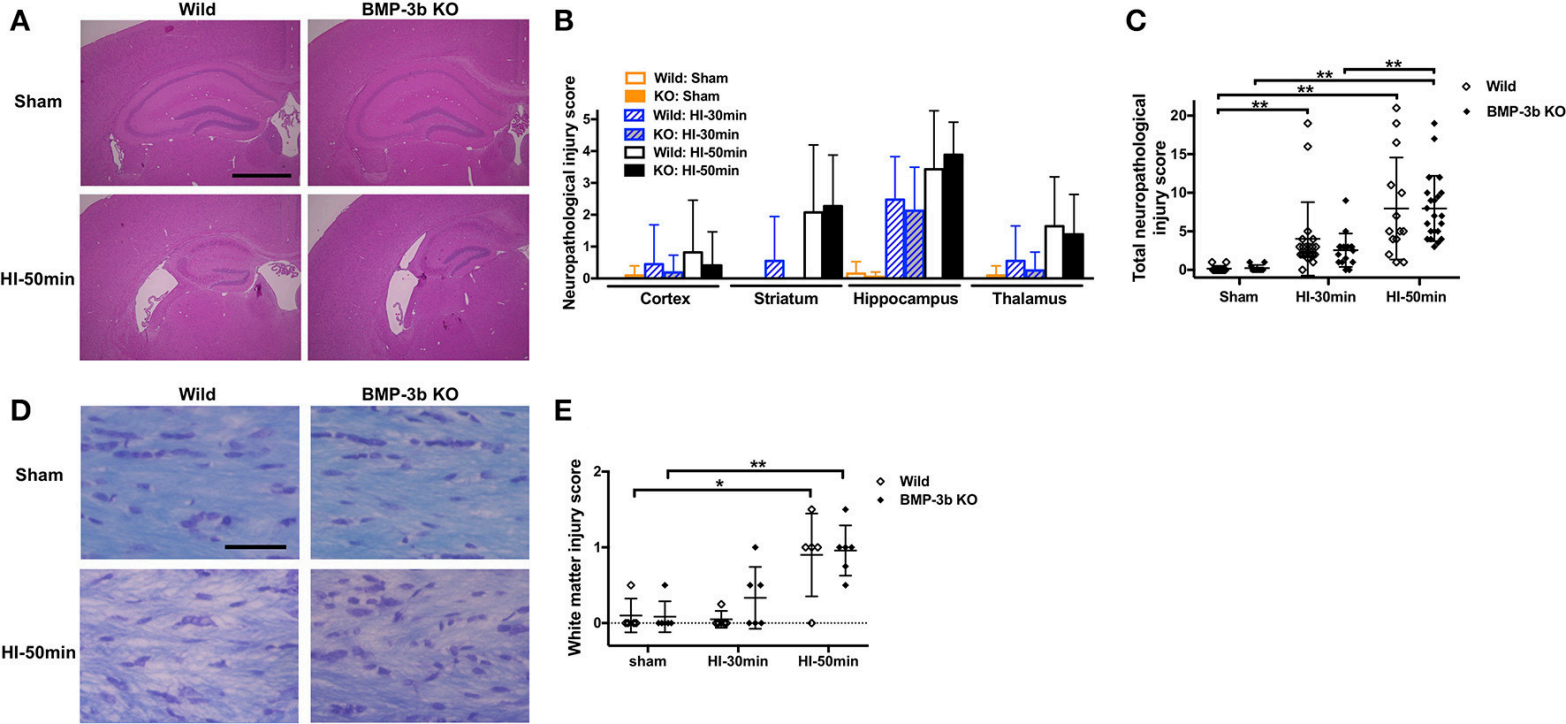
**(A)** Representative images of brain coronal sections stained with hematoxylin and eosin at 14 days after the surgery. Scale bar, 1 mm. **(B)** Semi-quantitative neuropathological injury scores were evaluated in hematoxylin-eosin-stained sections. The scores in each region are shown. Mean ± SD. Significant differences between groups treated with different degrees of insult are not shown with symbols in this figure. **(C)** The total scores of the four regions examined. The maximum (i.e., worst) score was 22. Wild Sham, *n* = 12; Wild HI-30 min, *n* = 20; Wild HI-50 min, *n* = 14; BMP-3b KO Sham, *n* = 12; BMP-3b KO HI-30 min, *n* = 16; and BMP-3b KO HI-50 min, *n* = 22. **(D)** Representative images of the Klüver–Barrera staining of the medial part of the corpus callosum. Scale bar, 50 μm. **(E)** Semi-quantitative white matter injury scores were evaluated in Klüver-Barrera-stained sections. The maximum (i.e., worst) score was 3. Each group, *n* = 5. ^*^p < 0.05, ^**^p < 0.01.

Similar to the previous reports, we did not find any macroscopic brain anomaly or sign of a specific disease in BMP-3b KO mice with sham-surgery. The HI-30 min and HI-50 min insults caused neuropathological injury in animals examined at 14 days after insult (Figures 7A,D). Neuropathological injury evaluated in hematoxylin-eosin stained sections was not significantly different between the wild type and BMP-3b KO groups whether they were either analyzed according to brain region (i.e., the cerebral cortex, striatum, hippocampus, and thalamus) (Figures 7A,B) or by total injury score (Figure 7C). White matter injury evaluated in sections with Klüver-Barrera staining was not significantly different between the wild type and BMP-3b KO groups (Figure 7E). There was no significant sex-dependent difference.

### Spleen weight

**Figure 8:**
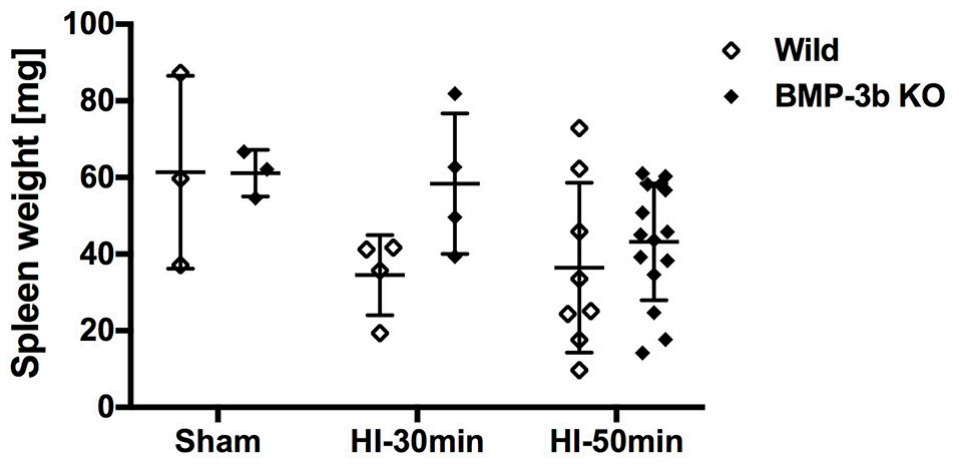
Spleen weights at 14 days after surgery. Wild Sham, *n* = 3; Wild HI-30 min, *n* = 4; Wild HI-50 min, *n* = 8; BMP-3b KO Sham, *n* = 3; BMP-3b KO HI-30 min, *n* = 4; and BMP-3b KO HI-50 min, *n* = 15.

Spleen weight is an indicator of inflammation and was similar between the wild type and BMP-3b KO pups at 14 days after sham-surgery (P26). Both HI insults reduced spleen weight (two-way ANOVA) (Figure 8). However, *post*-*hoc* tests did not reveal any significant differences according to genotype or the magnitude of the insult.

## Discussion

This is the first report to describe the phenotypes of BMP-3b/GDF10 KO mouse pups with brain injury. In sham-surgery-treated animals, BMP-3b gene depletion caused no changes in any parameter measured in this study except for hypoactive rearing in females. BMP-3b gene depletion resulted in (1) a striking increase in mortality in males treated with severe HI insult, (2) significantly hyperactive locomotion in males treated with severe HI insult, and (3) significantly hyperactive rearing in males and females treated with mild HI insult. However, BMP-3b gene depletion did not affect the results of any of the other tests (i.e., CBF, cylinder test, rotarod test, body weight gain, brain weight, spleen weight, % cerebral hemispheric volume loss, and semi-quantitative neuropathological injury score in four brain regions and white matter injury score). The most prominent finding in the current study was that BMP-3b may play a crucial role in helping animals survive severe HI insult.

During the last 15 min of the 50 min systemic hypoxic exposure period used in this study, the majority of BMP-3b KO male pups had a low rate of respiration, and respiration was shallow, ultimately leading to respiratory arrest. The specific mechanisms that lead to death during and immediately after HI insult are difficult to analyze in immature mice because physiological parameters, such as blood pressure, cannot be measured in these animals, especially during exposure to hypoxia. The mouse pups used in this study were subjected to unilateral carotid artery ligation followed by temporal systemic exposure to hypoxia. Hence, the HI insult was far more severe in the brain than in other organs, including the heart. The brain injury induced in this model was limited to the ipsilateral cerebral hemisphere, particularly the region that receives its blood flow from the middle cerebral artery. We hypothesize that brainstem dysfunctions inflicted by this type of cerebral injury may have been the cause of the observed deaths.

HI insult caused significantly more severe brain damage in the survivors with BMP-3b gene depletion than in those without the gene depletion. The BMP-3b KO pups were more hyperactive than the wild type pups were. These results suggest that BMP-3b plays a role in protecting against HI-induced brain damage and/or promoting recovery from this type of damage in survivors. We had expected that loss of BMP3b was more markedly detrimental to the brain both morphologically and behaviorally after the HI injury as suggested by the substantially high mortality during hypoxia in BMP3b KO mice. However, the high mortality in the KO group (as high as 70% in males) may have skewed the results, as only the resilient top 30% of pups subjected to the HI insult survived and were assessed. We considered that was the reason why the difference between wild type and BMP3b KO mice were not so remarkable and limited to only some of evaluations we performed.

No previous study has examined the role of BMP-3b after a brain injury except for one study performed by Li et al. (16) that evaluated adult stroke. They showed that BMP-3b expression is induced in peri-infarct neurons in mice and humans. They also demonstrated that BMP-3b induces axonal sprouting and enhances functional recovery after stroke in rodents and that knocking down BMP-3b blocks axonal sprouting and reduces recovery. Our results are basically in line with theirs. We found that suppressing the expression of the BMP-3b gene is deleterious to ischemic brain injury. However, the deleterious effects of BMP-3b gene suppression seemed to be more consistent and profound in their study. It may not be possible to directly compare the data from two such different studies. Several potential reasons for the observed differences are conceivable. For instance, (1) the ages of the models were different in that they used adult animals whereas we used immature pups; (2) the pathophysiology of the models was different in that theirs was stroke with photothrombosis whereas ours was HI; and (3) because the mortality rate observed in our study was high, it is possible that only inherently resilient sur‘rs remained for evaluation (the mortality rate in the BMP-3b knock-down used in the report by Li and colleagues was not provided). Despite the fact that cerebral ischemia was the main cause of injury in both models, neonatal HIE and adult stroke involve very different conditions from a number of standpoints, including their mechanisms of disease progression, clinical symptoms, and outcomes. As is the case in adult stroke patients, a photothrombosis model generally involves the induction of clearly demarcated and small ischemic lesions in the cerebral cortex with no or little reperfusion. In contrast, as has been observed in human newborns with HIE, the effects of HI in neonatal models affect most parts of the cerebral hemisphere and reperfusion proceeds immediately after the end of hypoxic exposure. The results of the present study support the notion that preclinical studies should be performed in an animal model appropriate to the disease.

To the best of our knowledge, this is the first report that revealed the loss of BMP-3b affected the survival rate after injuries. Hence, there is no study that BMP-3b was used to revert the mortality after brain injuries or other types of insult. The functional roles of BMP-3b in events following injuries/diseases remain largely unknown, although recent studies have gradually begun to unveil them. For example, BMP-3b selectively activates TGF-β receptor (TGF-βI/II)-dependent Smad3 phosphorylation and attenuates tumor formation (24). BMP-3b also increases energy expenditure and protects high-fat diet-induced obesity by suppressing peroxisome proliferator-activated receptor γ (PPARγ) (25).

The roles of other BMPs in the events involved in cerebral ischemia have been explored in several studies. The results of these studies suggest that BMPs may play detrimental roles in ischemic brain injury. For example, Dizon and colleagues reported that the levels of BMP-4 protein increase following HI insult in neonatal mouse pups and that inducing the transgenic expression of noggin, a BMP antagonist that binds BMP-2/4 with high affinity, confers a survival advantage (e.g., 90% of noggin-overexpressing pups and 59% of wild type pups survived) and ameliorates HI-induced white matter injury (8). BMP-3b and BMP-2/4 are mutually antagonistic (12), and the results of this previous study showed that antagonizing BMP-2/4 enhanced survival in neonatal HI, in line with our results. These data suggest that endogenous BMP-3b may enhance survival in neonatal HI. Using a different model of adult mice with permanent middle cerebral artery occlusion (MCAO), the same laboratory reported that noggin overexpression increased the number of PDGFRα^+^ cells (oligodendrocyte progenitor cells) in the ischemic boundary zone and ameliorated brain injury (7). Conversely, other studies have suggested that BMPs may exert beneficial roles in ischemic brain injury. For example, Wang and colleagues reported that the intracerebrally administering BMP-6 before inducing transient MCAO ameliorated brain injury in adult rats (5). The same group reported that BMP-7 signaling was increased in the brain after transient MCAO and that intravenously administering BMP-7 after inducing transient MCAO improved brain injury in adult rats (6). Treatment with BMP-7 did not change the mortality rate. Another group reported that the intracisternal administration of BMP-7 enhanced functional recovery in a rat model of stroke (4). Several possible factors may explain the seemingly contradictory effects of BMPs. For example, the ligands BMP-4 and BMP-6/7 have different preferred receptors.

The results of our study clearly demonstrate that BMP-3b protects immature mice from severe HI insult. Further studies are warranted to explore the precise mechanism of BMP-3b' beneficial effect and the therapeutic potential of using BMP-3b supplementation in neonatal HIE.

## Author contributions

YO performed the experiments (behavioral tests, etc.), analyzed the data, and prepared the figures. MT designed the study, performed the experiments (animal surgeries, etc.), and wrote the manuscript. ET performed the experiments (behavioral tests, etc.). JH designed the study, prepared the animals and critically revised the manuscript. MM critically revised the manuscript for important intellectual content. All authors gave their approval to the manuscript.

### Conflict of interest statement

The authors declare that the research was conducted in the absence of any commercial or financial relationships that could be construed as a potential conflict of interest.
